# Effects of polysaccharide from *Physalis alkekengi* var. *francheti* on liver injury and intestinal microflora in type-2 diabetic mice

**DOI:** 10.1080/13880209.2017.1345953

**Published:** 2017-08-23

**Authors:** Xin Zhao, Ziyang Chen, Yuling Yin, Xinli Li

**Affiliations:** Department of Biotechnology, Dalian Medical University, Dalian, P.R. China

**Keywords:** Antidiabetic effects, dominant organisms, protein expression, diabetic liver injury

## Abstract

**Context:** Diabetic liver injury is a serious diabetic complication. The alterations of intestinal microbiota play an important role in induction and promotion of liver injury progression. *Physalis alkekengi* L. var. *francheti* (Mast.) Makino (Solanaceae) has been used as a water decoction for treating diabetes.

**Objective:** To study the effects of a polysaccharide (PPSB) from *Physalis alkekengi* var. *francheti* on liver injury and intestinal microflora in type-2 diabetic mice.

**Materials and methods:** Streptozotocin (160 mg/kg) was injected i.p. for 3 days to build model. The diabetic mice were randomly divided into four groups together with control group (10 mice in each group). The doses of PPSB were 50 and 100 mg/kg, respectively. After 5 weeks administration, level of blood glucose, ALT and AST were measured. Alterations of intestinal microflora, and protein expression of TGF-β1, TNF-α and DCN were detected.

**Results:** Level of blood glucose decreased from (25.38 ± 2.21) mmol/L to (18.01 ± 2.53) mmol/L, ALT and AST decreased to (24.67 ± 4.86) U/L and (30.84 ± 7.50) U/L in PPSB-H group. *Lactobacillus*, *Clostridium butyricum*, and *Bacteroides* increased remarkably with increasing concentration of PPSB, but *Enterobacter* was inhibited. The relative expression of TGF-β1 and TNF-α decreased to (0.70 ± 0.17) and (0.39 ± 0.06), and the expression of DCN increased to (0.65 ± 0.13).

**Discussion and conclusions:** Probiotics have been promoted by PPSB, and protein expressions have been modulated in the progression of liver injury. PPSB could be used as a natural agent for treating diabetic liver injury and intestinal microflora imbalance.

## Introduction

Diabetes is a serious endocrine disorder causing morbidity and mortality worldwide. It has become the 3rd most common chronic disease harmful to human health, after tumours and cerebrovascular diseases. Indeed, according to the recent Diabetes Atlas produced by the International Diabetes Federation (IDF), China is home to the largest number of people with diabetes in the world, 109.6 million diabetic subjects were found in China in 2015, and these numbers are predicted to increase to 150.7 million by 2040. Diabetes needs long-term control by drugs; its increasing prevalence has put a large burden on society (Zhao et al. 2012). The chemical drugs used have some serious adverse reactions, such as hypoglycaemia, anaphylactic shock, liver damage, and cardiovascular toxicity (Xiao et al. 2011). The natural extracts/components have been paid more attention due to the fewer side effects or no harm to human bodies.

It has been reported that about 800 plant species possess the most antidiabetic potential, including *Physalis alkekengi* L. var. *francheti* (Mast.) Makino (Solanaceae). It is a well-known edible and medicinal plant in the northeastern part of China. Studies on the potential biological functions of active components such as physalins have attracted more attention in recent years (Ji et al. 2012). Another active component, polysaccharide fraction (PPSB) from fruit calyx, could be used as a therapeutic agent for scavenging free radical (Ge et al. 2009) and reducing blood glucose levels and water intake (Tong et al. 2011). Our previous study (Li et al. 2014) also showed that the intestinal microflora imbalance caused by antibiotic could be improved by PPSB.

Diabetes is estimated to be the most common cause of liver diseases (Wan et al. 2016). At the early stage of diabetic liver injury (DLI), clinical symptoms were unobvious which is due to the powerful compensatory function of liver. Moreover, once the patients developed liver fat deposition and fibrosis, the structure and function of liver were susceptible to damage (Domitrovi 2011). Fibrotic process of the liver begins from lipid peroxidation and accumulation of extracellular matrix (ECM) including collagen, proteoglycan, and adhesive glycoproteins, which are principally produced by activated hepatic stellate cells (HSCs). Transforming growth factor-β1 (TGF-β1) increases the accumulation of these matrix proteins at the injury site (Goetsch and Niesler 2016) and promotes fibrosis, whereas the proteoglycan decorin (DCN) is known to act as an antifibrotic agent, in part via the binding and neutralization of TGF-β1. Accumulating evidences have demonstrated that natural extracts possessed the ability to prevent liver fibrosis by reducing the expressions of TGF-β1 and tumour necrosis factor-α (TNF-α), and increasing the expression of DCN in the liver (Thu et al. 2016).

Ruth et al. (2006) and Cani and Delzenne (2009) revealed that intestinal microflora play an important role in the modulation of intermediary phenotypes leading to metabolic disease in addition to the genetic and environmental factors. In our preliminary study (Wang et al. 2016), *Lactobacillus* and *Bacteroides* decreased remarkably in type-2 diabetic (T2D) mice. There is a strong relationship between liver and gut: the portal system receives blood from the gut, and intestinal blood content activates liver functions. The liver, in turn, affects intestinal functions through bile secretion into the intestinal lumen. But, to the best of our knowledge, the relationship between changes of intestinal microflora and the degree of liver injury in T2D has not been carried out. We therefore specifically used a streptozotocin-induced diabetic mice model to evaluate the effects on the intestinal microflora and examine the mechanism of hepatoprotective activity of PPSB by the expressions of TNF-α, TGF-β1, and DCN, furthermore try to explore the association between intestinal microflora and liver injury in T2D.

## Materials and methods

### Plant material and reagents

The calyces of *P. alkekengi* L. var. *francheti* (Mast.) Makino were purchased from Dalian Traditional Chinese Medicine Market in October 2011 and identified by Professor Yuling Yin (Dalian Medical University) according to the standard of Pharmacopeia of the People’s Republic of China. A voucher specimen (No. LP201101) is deposited in Department of Biotechnology, Dalian Medical University, China.

PPSB has been isolated and characterized in the previous experiment (Li et al. 2014). Streptozotocin was purchased from Sigma Chemical Co. (St. Louis, MO). Dimethylbiguanide (purity ≥97%) was purchased from Beijing Zhonghui Drug Co. (Beijing, China). Blood glucose kit was purchased from Dalian Chenyu Biotechnology Co., Ltd. (Dalian, China). Stool DNA kit was purchased from ForeGenen (Chengdu, China). Polymerase chain reaction primers GC-357f (CGCCCGGGGCGCGCCCCGGGCGGGGCGGGGGACGGGGGGCCTACGGGAGGCAGCAG), 518r (ATTACCGCGGCTGCTGG), and 357f (CCTACGGGAGGCAGCAG) (Muyzer et al. 1993) were synthesized by TaKaRa Biotechnology Co., Ltd. (Dalian, China). PCR Mix was purchased from Beijing TransGen Biotech Co., Ltd. (Beijing, China). Antibodies against TNF-α, TGF-β1, DCN, β-actin, and HRP-conjugated AffiniPure goat anti-rabbit IgG (H + L) were obtained from Proteintech Group Inc. (Chicago, IL). The enhanced chemiluminescence (ECL) kit was from Amersham Life Science, Inc. (Arlington Heights, IL). Alanine aminotransferase (ALT) and aspartate aminotransferase (AST) were purchased from the Jiancheng Bioengineering Institute (Nanjing, China). All other chemical reagents used were analytical grade.

### Experimental animals

Male KM mice weighing 30 ± 3 g, purchased from animal experimental centre of Dalian Medical University [Certificate of quality number: SCXK (Liao) 2013-0003], were used. The mice were kept under standardized conditions at a temperature of 22–24 °C, and 20% humidity with a 12-h light/dark cycle, and they had free access to standard diet and water *ad libitum*. They were allowed to acclimatize for five days before the experiments were started.

### Streptozotocin-induced diabetic model in mice

A freshly prepared solution of streptozotocin (160 mg/kg) in 0.1 M citrate buffer (pH 4.2) was injected i.p. to the overnight fasted mice. The blood glucose (BG) level was determined at the 3 days after injection. The mice with the levels of blood glucose ranging from 10 to 25 mM were considered as the successful experimental animals of T2D model and used for the further study (Umar et al. 2010).

### Experimental design

The streptozotocin-induced diabetic mice were randomly divided into four groups (Groups 2–5) together with blank control group (Group 1) (10 mice in each group). They were administered for five weeks, and the arrangements are as follows:

Group 1: Blank control group (N), normal mice treated with water.
Group 2: Diabetic model group (D), diabetic mice with distilled water.
Group 3: Diabetic mice with 600 mg/kg of dimethylbiguanide (E) (Yang et al. 2008).
Group 4: Diabetic mice with 50 mg/kg of PPSB (L).
Group 5: Diabetic mice with 100 mg/kg of PPSB (H).

All groups were treated by intragastric administration once a day for five weeks. The consumption of diet and water, and body weight were recorded daily. The level of BG was directly measured by blood glucose kit after the administration each week. After 5 weeks’ treatment, faeces and liver of each mouse were collected and preserved at −80 °C.

### Biochemical analysis

Level of BG was measured by blood glucose kit. ALT and AST were estimated according to the Reitman-Frankel method.

### Deoxyribonucleic acid (DNA) extraction

DNA was extracted from faecal samples with Stool DNA kit in accordance with the manufacturer’s instructions. The amount and quality of DNA extracts were analyzed by electrophoresis of 1% agarose gel containing ethidium bromide and compared to a molecular weight standard (1000 base pairs, bp). The DNA concentration was measured spectrophotometrically using NanoVue Biophotometer Plus (Bio-Rad, Hercules, CA) and DNA extracts were preserved at −20 °C until used.

### Polymerase chain reaction (PCR) amplification

Primers GC-357f and 518r were used to amplify the V3 region of bacterial 16S rRNA. PCR amplification was performed with an automated thermocycler (Thermo Fisher Scientific, Waltham, MA) as follows: 2 μL purified genomic DNA as template, 2 × Easy Taq PCR SuperMix 12.5 μL, forward primer (10 μM) 0.5 μL, reverse primer (10 μM) 0.5 μL, and filled up to a volume of 25 μL with sterile Milli-Q water. The thermal program consisted of an initial denaturation step of 94 °C for 5 min, followed by 30 cycles of 94 °C, 54 °C, 72 °C for 30 s each, in which the annealing temperature 72 °C for 7 min. Amplification products were analyzed first by electrophoresis of 1% agarose gel containing ethidium bromide and compared to a molecular weight standard (100 bp).

### DGGE analysis

The PCR products were electrophoresed on 8% polyacrylamide (acrylamide/bisacrylamide, 37.5:1) gels containing a linear denaturant gradient ranging from 25 to 50%, with 100% denaturant defined as a solution of 7 M urea and 40% (*v/v*) deionized formamide. Electrophoresis was performed, first for 10 min at 200 V, and subsequently for 16 h at 70 V in a 1 × TAE buffer at a constant temperature of 60 °C. Gels were stained with AgNO_3_.

### Sequence analysis

To identify some separated and strong bands, a sterile scalpel was used to cut out the bands from polyacrylamide gel. Gel fragments were eluted in 20 μL sterile water at 4 °C overnight. The eluted DNA (4 μL) was reamplified by PCR following the program described previously, and only the forward primer was 357f. Each PCR product was also subjected to DGGE analysis to confirm whether the band was purified or not. Subsequently, idiographic sequences were attained by TaKaRa Biotechnology (Dalian) Co., Ltd. Finally, the sequences were compared directly with those in GenBank by BLAST search (http://blast.ncbi.nlm.nih.gov/Blast.cgi).

### Western blot analysis

Total protein was extracted from the liver samples using RIPA lysis buffer with protease inhibitors in a proportion of 1:100. The BCA assay kit was used to quantitative protein. Equal amounts of protein (120 μg) were separated by 10% SDS-PAGE gel using 100 V for 3 h and then transferred to nitrocellulose membrane by semidry apparatus for 50 min for β-actin, 25 min for TNF-α, 30 min for TGF-β1, and 50 min for DCN, respectively. The membranes were blocked with 5% nonfat milk for 2 h at room temperature and then incubated with primary antibodies against TNF-α, TGF-β1, DCN, and β-actin, respectively, at a 1:500 dilution overnight at 4 °C. The next day, the membranes were incubated with secondary antibody at a 1:2000 dilution for 2 h at room temperature after washed with TBST three times. Then, the protein bands were visualized using ECL kit by Bio-Rad ChemiDoc XRS plus image analyzer (Bio-Rad, Hercules, CA) after TBST washing as previously described. β-Actin was used as internal reference (Jiang et al. 2016).

### Statistical analysis

The statistical software SPSS version 17.0 was used for analysis (Chicago, IL). *p*-Values were determined using the *t*-test, and *p*-value <0.01 was considered significant. DGGE and Western blot gels were analyzed by using Quantity One 4.6.2 gel analysis software (Bio-Rad, Hercules, CA). Similarities were displayed graphically as a dendrogram. The clustering algorithms used to calculate the dendrograms were an unweighted pair group method with arithmetic average (UPGMA). The Shannon–Wiener index of diversity (*H′*) was used to determine the diversity of the bacterial community. The evenness (E) which reflected uniformity of bacterial species distribution was also computed.

## Results

### Isolation and purification of PPSB

PPSB has been isolated and characterized in the previous experiment (Li et al. 2014). The crude polysaccharide from the calyces of *P. alkekengi* var. *francheti* was extracted by hot water and ethanol precipitation with a yield of 13.6%. After deproteinated by a combination of proteinase and Sevag method, the crude polysaccharide sample was purified by DEAE-52 Cellulose and Sephadex G-200 gel column to obtain PPSB fraction.

### Physicochemical properties and chemical compositions

PPSB appeared as a white powder and had a negative response to Bradford assay. Phenol–sulphuric acid assay showed PPSB contained 92.5% carbohydrate. No absorption at either 260 or 280 nm was detected by UV spectrophotometer. The molecular weight of PPSB was evaluated by HPLC equipped with a TSK gel G3000 PWXL column (7.8 × 300 mm) and a RID-10 A refractive index detector. HPLC was performed on 0.5% MSP (20 μL) dissolved in distilled water with 0.7% Na_2_SO_4_ as the mobile phase at 0.5 mL/min and 35 °C. The results showed that PPSB (*M*_w_ = 27 kDa) which is acid heteropolysaccharide consists of Ara, Gal, Glc, and GalA in the ratio of 2.6:3.6:2:1 (Tong et al. 2008).

### Effects of PPSB on BG

**Figure 1.**
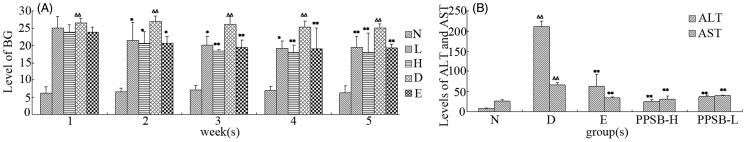
Levels of blood glucose (A), ALT and AST (B) of normal and streptozotocin-induced diabetic mice (X¯ ± s, *n* = 10). ^ΔΔ^*p* < 0.01, ^Δ^*p* < 0.05 vs. the blank control group (N); **p < 0.01, *p < 0.05 vs. the diabetic model group (D). N: normal mice treated with water; L: diabetic mice with 50 mg/kg of PPSB; H: diabetic mice with 100 mg/kg of PPSB; D: diabetic mice with distilled water; E: diabetic mice with 600 mg/kg of dimethylbiguanide.

In the experiments, streptozotocin-induced diabetic mice showed typical symptoms of mental depression, lustreless body complexion, and slow response. As shown in Figure 1(A), the level of blood glucose in group N maintained constant for five weeks and was significantly (*p* < 0.01) lower than those of streptozotocin-induced diabetic mice of the remaining four groups. That suggested streptozotocin-induced diabetic model in mice has been established successfully. Compared to group D, the level of BG in group H decreased regularly from (25.38 ± 2.21) mmol/L to (18.01 ± 2.53) mmol/L during five weeks, which showed extremely significant hypoglycaemic effects (*p* < 0.01) at the 3rd week. In addition, the significant difference in group L and E was shown from the 2nd to 5th weeks.

### Effects of PPSB on ALT and AST

ALT and AST in the serum have been commonly used as biochemical markers for liver injury. The significantly elevated AST and ALT expression levels indicated the increased permeability and damage of liver. In the present study (Figure 1(B)), the ALT and AST serum concentrations were increased to (211.07 ± 13.72) U/L and (65.70 ± 6.26) U/L in streptozotocin-induced diabetic mice group, which indicated streptozotocin had severely damaged liver tissues. Interestingly, significant decreases in ALT and AST were observed in E and PPSB-treated groups when compared with D group. ALT and AST decreased to (24.67 ± 4.86) U/L and (30.84 ± 7.50) U/L in PPSB-H-treated group, respectively.

### DGGE analysis

**Figure 2.**
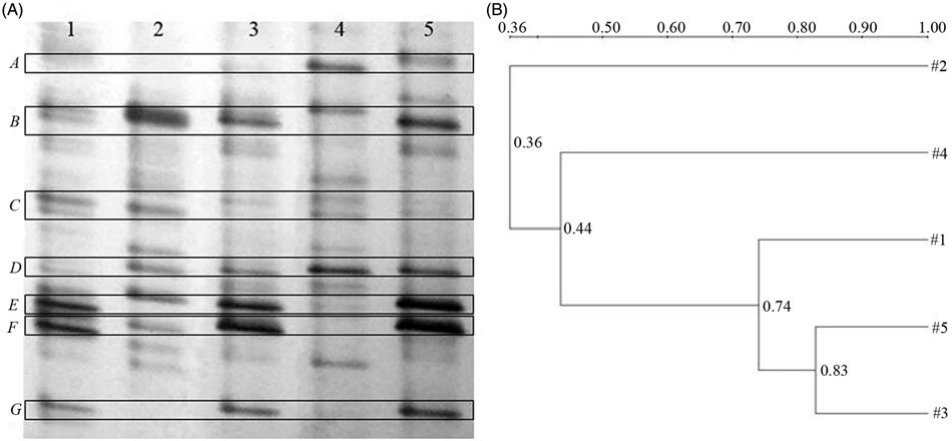
Representative DGGE profiles (A) and UPGMA dendrograms (B) of blank control, diabetic mice, diabetic mice with 50 and 100 mg/kg of PPSB, and positive control groups. Group 1: Blank control group (N), normal mice treated with water. Group 2: Diabetic model group (D), diabetic mice with distilled water. Group 3: Diabetic mice with 600 mg/kg of dimethylbiguanide. Group 4: Diabetic mice with 50 mg/kg of PPSB (L). Group 5: Diabetic mice with 100 mg/kg of PPSB (H).

The dominant intestinal microflora of group N, L, H, D, and E was examined by DGGE analysis with universal primers targeting the V3 region of the 16S rRNA (Figure 2(A)). Lane 1 was the sample from blank normal group, while lane 2 represented those from diabetic model group, lane 3 represented those from streptozotocin-induced diabetic mice with 600 mg/kg of dimethylbiguanide, and lanes 4 and 5 represented those from streptozotocin-induced diabetic mice with 50 and 100 mg/kg of PPSB, respectively. Obviously, *B* was found in streptozotocin-induced diabetic mice groups (Group 2–5), the intensity of the band was highest in D group especially, but it did not exist in normal group. *A* and *D* increased remarkably in PPSB-L and PPSB-H groups. *E*, *F*, and *G* existed in normal, dimethylbiguanide-treated, and PPSB-H-treated mice groups, but almost disappeared in other two groups. Other bands almost existed in all groups, and in the different groups, the intensity of the bands in the same position was similar.

The clustering analysis based on the values of Dice coefficients was visualized in an UPGMA dendrogram to study general patterns of community similarity among the different groups by Quantity One software. Figure 2(B) displayed that different groups formed the statistically significant clustering profiles. There were three main clusters in the dendrogram, the first was lane 2 related D group, the second was lane 4 related PPSB-L group, and the 3rd was lanes 1, 3, and 5 related N, E, and PPSB-H groups. The maximum bacterial similarity index between PPSB-H and E groups was 0.83, and D group possessed the lowest similarity (0.36) to other groups, which suggested that the intestinal microflora community of streptozotocin-induced diabetic mice was affected obviously, but that of PPSB-treated mice with 100 mg/kg and dimethylbiguanide-treated mice were similar. In addition, Figure 2(B) showed that the similarity among E, PPSB-H and N groups were high (0.74), which suggested that the effects of dimethylbiguanide and PPSB (100 mg/kg) on intestinal microflora community were similar to normal.

DGGE profiles displayed the typical characteristics of general bacteria in the intestinal tract. Each band derives possibly from one phylogenetically distinct community; hence, an estimation of species number could be based on the total number of the bands in the profile. The Shannon–Wiener indexes of *H′* reflecting the structural diversity of the bacterial community were calculated on the basis of the number and relative intensities of bands on the gel (Table 1).

**Table 1.** Microflora diversity index analysis^a^ (X¯ ± s, *n* = 10).

Group	S	*H′*^b^	E^c^
N	15.00 ± 0.82	1.9104 ± 0.0321	0.7061 ± 0.0241
D	12.75 ± 1.26*	1.3067 ± 0.0104**	0.5148 ± 0.0231**
E	13.00 ± 0.82*	0.8667 ± 0.0206**	0.3382 ± 0.0101**
L	10.00 ± 0.82**	1.8825 ± 0.0208	0.8192 ± 0.0207**
H	12.25 ± 1.26*	0.8351 ± 0.0063**	0.3342 ± 0.0112**

N: normal mice treated with water; D: diabetic mice with distilled water; E: diabetic mice with 600 mg/kg of dimethylbiguanide; L: diabetic mice with 50 mg/kg of PPSB; H: diabetic mice with 100 mg/kg of PPSB.

^a^** *p* < 0.01 vs. the blank control group (N).

b *H′* = −∑ (*p_i_*) (ln*p_i_*), where *p_i_* was the proportion of the bands in the track, and *p_i_*= *n_i_*/∑*n_i_*, where *n_i_* was the average density of peak *i* in the densitometric curve.

^c^E = *H′*/ln S, where S was the number of bands.

It was clearly shown that diversity in PPSB-L, PPSB-H, D, and E groups decreased as compared to N group with statistical significance (*p* < 0.05). Compared to N group, the numbers of bands were lower in streptozotocin-induced diabetic groups, and they all showed significant (*p* < 0.01) differences. Especially D, E, and PPSB-H groups produced low evenness score, but PPSB-L showed relatively high score, apparently due to a richer numbers of intense bands in the above three groups. Based on these results, it appeared that intestinal microflora was changed by streptozotocin seriously, and the richness (S), diversity index (*H*′), and evenness score (E) decreased.

Data in Table 2 showed the closest relatives based on the results of BLAST searches with DNA sequences obtained from DGGE gel bands identified by cluster analysis. Bands in the same position but in different lanes were excised and sequenced to confirm that they had the same identity (data not shown). *E* and *F* were sequenced and identified as *Lactobacillus johnsonii* and *Lactobacillus amylolyticus* with the similarity of 97% and 98%, respectively. *G* was sequenced and identified as *Clostridium butyricum* str. with the similarity of 97%. *Lactobacillus* and *Clostridium* both existed in N, E, and PPSB-H groups, while there was nearly no band at the corresponding place from D group particularly. *A* and *D* were sequenced and identified as *Bacteroides* sp. and *Bacteroides ovatus* with the similarity of 100% and 99%, and they increased remarkably in PPSB-treated groups, but did not exist in other groups. *B* and *C* were sequenced and identified as *Enterobacter cloacae* and *Prevotella micans*, respectively, with the similarity of 99% and 97%. *Enterobacter* existed in streptozotocin-induced diabetic mice groups, as well as the intensity weakened with dimethylbiguanide and increasing concentration of PPSB.

**Table 2.** Sequences of PCR amplicons derived from DGGE gels and identities based on the BLAST database.

Selected band	Most similar sequence relative (GenBank accession number)	Bacteria genus	Identity (%)
*A*	*Bacteroides* sp. (ADCL01000128.1)	*Bacteroides*	100
*D*	*Bacteroides ovatus* (AAXF02000050.1)		99
*G*	*Clostridium butyricum* str. (ACOM01000003.1)	*Clostridium*	97
*E*	*Lactobacillus johnsonii* (AFQJ01000002.1)	*Lactobacillus*	97
*F*	*Lactobacillus amylolyticus* (ADNY01000006.1)		98
*B*	*Enterobacter cloacae* (NC 014618.1)	*Enterobacter*	99
*C*	*Prevotella amnii* (ADFQ01000002.1)	*Prevotella*	97

DGGE analysis indicated that the intestinal microflora of mice was changed by streptozotocin administration obviously. The bacteria from the genera of *Bacteroides*, *Lactobacillus*, *Enterobacter*, *Clostridium*, and *Prevotella* were dominant organisms in the intestinal tract of mice of different groups by DGGE analysis. We proposed that the change of dominant intestinal microflora plays a causal role in T2D, and specifically, *Lactobacillus*, *Clostridium*, and *Bacteroides* decreased but *Enterobacter* increased in streptozotocin-induced diabetic groups.

### Expression of TNF-α, TGF-β1, and DCN by PPSB

**Figure 3.**
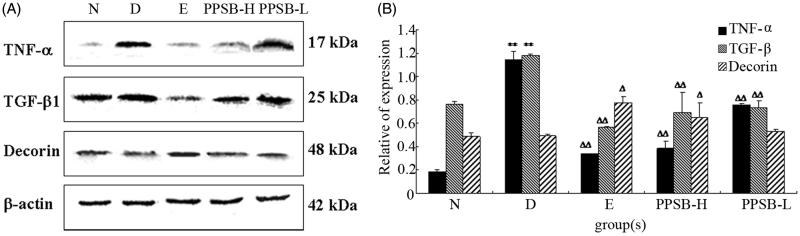
Effects of PPSB on expression of TGF-β1, TNF-α, and DCN in livers. **p < 0.01, *p < 0.05 vs. the blank control group (N); ^ΔΔ^*p* < 0.01, ^Δ^*p* < 0.05 vs. the diabetic model group (D). N: normal mice treated with water; L: diabetic mice with 50 mg/kg of PPSB; H: diabetic mice with 100 mg/kg of PPSB; D: diabetic mice with distilled water; E: diabetic mice with 600 mg/kg of dimethylbiguanide.

TGF-β1 and TNF-α have been implicated as important factors that are responsible for triggering and mediating the fibrotic responses. Inhibition of them is essential in therapy to reduce fibrosis (Mosca et al. 2015). Western blot analysis showed that dimethylbiguanide and PPSB treatment could dramatically decrease TGF-β1 and TNF-α expression levels in the liver of streptozotocin-induced diabetic mice. The relative expression of TGF-β1 and TNF-α in PPSB-H-treated group decreased to (0.70 ± 0.17) and (0.39 ± 0.06) (*p* < 0.01) (Figure 3). Oppositely, the expression of DCN increased to (0.65 ± 0.13). It suggested that the hepatoprotective activity of PPSB on diabetic liver injury was at least partially related to inhibit the expressions of TGF-β1 and TNF-α, and promote the expression of DCN.

## Discussion and conclusions

Treatment of diabetes remains a major therapeutic challenge that the entire population needs to pay attention. Although numerous hypotheses have been proposed to link it, no clear unifying concept has yet emerged. Intestinal microflora is determinant of body weight and the amount (size) of adipose tissue which has been evidenced in recent reports; it suggests that the microflora play an important role in the development of T2D. Intestinal microflora could be considered as a ‘microbial organ’ placed within a host organism. It exerts harmful and beneficial effects on human health. Cancer, inflammation, and metabolic diseases are among the first in line for the use of microflora-based therapeutic strategies (Serino et al. 2009).

The severity of diabetic liver injury (DLI) may be aggravated by T2D (Lee et al. 2017). Increasing numbers of reports (Cesaroa et al. 2011) have indicated that alterations of intestinal microbiota play an important role in induction and promotion of liver damage progression, in addition to direct injury resulting from different causal agents. Probiotics have been suggested as a useful integrative treatment of different types of chronic liver damage, for their ability to augment intestinal barrier function and prevent bacterial translocation.

*Lactobacillus* spp., *Bacteroides*, and *Clostridium butyricum* are probiotics, and they were antagonistic to pathogenic microorganisms of intestinal microflora, which could help host to decompose polysaccharide and improve the efficiency of nutrition (Bäckhed et al. 2004), increase lymphocyte numbers, speed up the vascularization of the gut mucosa (Stappenbeck et al. 2002), and modulate the gastrointestinal microflora (Kong et al. 2011). Together, the results obtained in rodents and humans demonstrated that obesity and type-2 diabetes can be associated with an altered gut microbiota. Compared to lean mice, genetically obese mice have 50% fewer Bacteroidetes, and a proportional increase in Firmicutes (Ruth et al. 2006).

In the present study, PPSBs as antidiabetic compounds could decrease the levels of blood glucose with significant difference (*p* < 0.01) and there was no significant difference when compared to positive control (600 mg/kg of dimethylbiguanide) group. On other hand, compared to D group, the ALT and AST of PPSB-treated groups decreased and showed extremely significant (*p* < 0.01) differences, respectively. The growth of *Lactobacillus*, *Clostridium butyricum*, and *Bacteroides* has been dramatically promoted by PPSB, but *Enterobacter* which was opportunistic bacterial pathogen has been inhibited that suggested an improvement occurred in the alteration of intestinal microflora. The expression levels of TGF-β1 and TNF-α decreased in PPSB-treated groups; in addition, the expression of DCN increased as compared to D group.

It is clear that side effects of T2D are associated with whole intestinal microflora and liver injury. With the increased dose of PPSB, *Lactobacillus*, *Clostridium butyricum*, and *Bacteroides* increased both in terms of quality and quantity, and it is in accordance with the characteristics of prebiotics. Meanwhile, probiotics in intestinal tract which promoted by PPSB also modulate several protein expressions (TGF-β1, TNF-α, and DCN) in the induction and progression of liver injury, and they play an important role. So PPSB could be considered as a potential candidate for developing a new agent which could treat diabetic liver injury and restore the intestinal microflora balance.
